# A retrospective cohort study of how alveolar ridge preservation affects the need of alveolar ridge augmentation at posterior tooth implant sites

**DOI:** 10.1007/s00784-021-03778-y

**Published:** 2021-01-11

**Authors:** Kai-Fang Hu, Ying-Chu Lin, Yu-Ting Huang, Yu-Hsiang Chou

**Affiliations:** 1 Ph.D. Program in Environmental and Occupational Medicine, College of Medicine, Kaohsiung Medical University and National Health Research Institutes, Kaohsiung, Taiwan; 2 Institute of Clinical Medicine, National Yang-Ming University, Taipei, Taiwan; 3 Division of Periodontics, Department of Dentistry, Kaohsiung Medical University Hospital, Kaohsiung, Taiwan; 4 School of Dentistry, College of Dental Medicine, Kaohsiung Medical University, No.100, Tzyou 1st Road, Kaohsiung, 807 Taiwan; 5 Division of Medical Statistics and Bioinformatics, Department of Medical Research, Kaohsiung Medical University Hospital, Kaohsiung Medical University, Kaohsiung, Taiwan

**Keywords:** Retrospective study, Implant, Alveolar ridge augmentation, Alveolar ridge preservation

## Abstract

**Objectives:**

The aim of this study was to assess whether alveolar ridge preservation (ARP) can reduce the need of ridge augmentation at posterior tooth sites.

**Material and methods:**

This study enrolled patients who received dental implants at posterior tooth sites during 2013–2019. Demographic data and dental treatment histories were collected. Based on healing patterns after tooth extraction, patients were divided into ARP and spontaneous healing (SH) groups. Three surgical treatment plans were devised according to the alveolar bone volume on cone-beam computed tomography (CBCT). The three treatment plans were to perform implant alone, simultaneous guided bone regeneration (GBR) and implantation, and staged GBR before implantation. Statistical analyses were performed to determine relationships.

**Results:**

There were 92 implant records in the ARP group and 249 implant records in the SH group. A significant intergroup difference was observed regarding the frequency distribution of the treatment modality of staged GBR before implant (*χ* ^2^ = 15.07, *p* = 0.0005). Based on the implant alone treatment modality and simple logistic regression, the SH pattern was related to staged GBR before implant (SH vs. ARP: crude odds ratio (OR) = 4.65, 95% confidence interval (CI) = 2.15–11.61, *p* = 0.0003). After adjusting confounding factors, the risk was still significant (adjusted OR = 5.02, 95% CI = 2.26–12.85, *p* = 0.0002).

**Conclusions:**

The study results suggested that ARP is more likely to lead to the treatment modality of implant alone and reduce the need for staged GBR before implantation.

**Clinical relevance:**

This study describes ARP capable of minimizing the need for staged GBR before implantation and shortening the treatment duration.

## Introduction

Alveolar ridge resorption after a tooth extraction is an inevitable phenomenon [1]. After tooth extraction, the bundle bone lining the alveolar socket loses its function and is absorbed [2]. Resorption of the alveolar ridges most often occurs during the first 6 months after the extraction [1, 3] and continues to decrease at an average rate of 0.25 to 0.5% per year until death [4]. By measuring study casts, linear radiographic analyses, and subtraction radiography, Schropp et al. estimated that two-third of hard and soft tissue changes occurred during the first 3 months and 50% of the crestal width was resorbed during 12 months [5].

Notably, the maximal bone loss occurs in the horizontal dimension, especially at the facial aspect of the ridge. In a systematic review article, Van der et al. proposed that the ridge resorption width is 3.87 mm [6]. Moreover, a loss of vertical ridge height occurs and is most pronounced at the buccal aspect [7, 8]. Furthermore, Van der et al. reported that the loss of bone height at the mid-buccal site is 1.67 mm [6]. In accordance with the findings of these studies, another systematic review described that horizontal alveolar ridge loss was more than vertical bone loss during the initial 6 months [9]. The recent systemic review found that ridge reduction after extraction at non-molar sites was 2.73 mm in clinically horizontal direction. The horizontal bone resorptions assessed radiographically in non-molar and molar sites were 2.54 mm and 3.61 mm, respectively [10].

Alveolar ridge atrophy may considerably affect tooth replacement therapies, particularly in dental implant-supported restorations [11]. Therefore, to reduce the loss of alveolar bone after tooth extraction, several surgical techniques have been proposed to maintain an ideal ridge profile, prevent alveolar ridge collapsing, preserve adequate dimensions of the bone, and facilitate correct dental implant placement [12]. Consequently, a concept called “alveolar ridge preservation” (ARP) or “alveolar bone grafting” was utilized to reduce the dimensional changes after tooth extraction. ARP is defined as “any procedure undertaken at the time of extraction that is designed to minimize external resorption of the ridge and maximize bone formation within the socket” [13]. The technique of ARP was via socket grafting and socket sealing. A systematic review by Avila-Ortiz and coworkers mentioned nine different ARP treatment modalities [14]. Numerous graft materials have already been investigated for ARP at the extraction site, including autogenous bone [15], demineralized freeze-dried bone allograft [16], xenografts [17], and alloplastic grafts [18]. Furthermore, various systematic reviews have been conducted to confirm the efficacy of ARP to prevent the post-extraction resorption of the alveolar bone [19–23] [14]. Vignoletti et al. indicated that the advantages of ARP included maintenance of the existing soft and hard tissue to optimize dental implant function, achieve esthetic demands, and simplify treatment procedures before dental implantation [24]. Most review articles have confirmed that ARP caused significantly less vertical and horizontal resorption of the alveolar bone at the crest level. However, until date, few studies have discussed the benefits of ARP, such as simplifying treatment procedures and averting alveolar ridge augmentation before dental implantation at extraction sites. Therefore, this study assessed the effects of ARP in reducing the need for alveolar ridge augmentation procedures before dental implantation.

## Materials and methods

### Study population

The study protocol was approved by the Institutional Review Board of the Kaohsiung Medical University Hospital (KMUHIRB-E (II)-20190433) and conformed to recognized standards of Declaration of Helsinki. We enrolled consecutive patients who received dental implant treatment at the Division of Periodontics, Kaohsiung Medical University Hospital, during 2013–2019. Patients with an anterior tooth dental implant, aged less than 20 years, or on oral and high-dose intravenous administration of bisphosphonates were excluded from this study. We collected past dental histories and clinical treatment records at the dental implant sites of these patients. Demographic data included gender, age at treatment, dental implant position (dental arch and tooth site), reasons for tooth extraction, systemic diseases, and smoking status. The reasons for tooth extraction were classified as periodontitis and non-periodontitis. Reasons for tooth extractions in the non-periodontitis group were crown or root fracture, deep caries, chronic apical periodontitis, and malposition. Systemic diseases were hypertension, diabetes mellitus, and hepatitis. Smoking status was classified as former smokers and non-smokers. The healing patterns of tooth extraction sites were divided into ARP and spontaneous healing (SH) groups. The past dental treatment records showed that ARP was immediately performed after the tooth extraction by filling the extracted socket with freeze-dried bone allograft (FDBA, LifeNet Health®) and coronal covering with platelet-rich fibrin (PRF). If no additive substance was placed into the extracted socket, the healing patterns of the tooth extraction site were defined as SH.

### Surgical treatment plan of the dental implant site

A prosthodontic-driven surgical stent indicating the future dental implant position was placed at tooth extraction sites before patients received a cone-beam computed tomography (CBCT) examination. In the ARP group, CBCT was performed 4–5 months after extraction. In the SH group, CBCT was performed when the patient wanted to receive dental implant therapy. The surgical treatment plan of dental implant placement was designed according to the alveolar bone width calculated based on CBCT images. The treatment modalities were classified into three types. (A) When alveolar bone width from buccal to lingual or palatal side at the crestal level was less than 4 mm or severe bone resorption was observed at either side, a surgical treatment modality of bone grafting augmentation procedure via GBR prior to dental implant placement was performed. (B) When the alveolar bone width at the crestal level ranged from 4 to 6 mm or moderate bone resorption was observed at either side, bone grafting augmentation procedure via GBR at the same time of dental implant placement was performed. (C) When the alveolar bone width at the crestal level was > 6 mm from buccal to lingual or palatal aspects and no bone resorption or undercut was observed at either side, a dental implant placement without ancillary bone grafting augmentation procedure was followed.

### Statistical analysis

Numerical data in the ARP and SH groups are presented as the mean ± standard deviation (SD), and frequency distributions within each group are indicated with numbers and proportions. The chi-square test and two-sample *t* test were used to compare intergroup differences in distribution proportions and means, respectively.

The effects of healing patterns of the extraction socket on various treatment modalities were determined using simple logistic regression, and their effect size is indicated as the odds ratio (OR) with a 95% confidence interval (CI). Multivariate logistic regression was performed to assess the relationship after adjusting for the potential confounding factors of gender, age at treatment, arch, site, reasons for tooth extraction, and smoking status. Statistical significance was set at *p* < 0.05. All statistical analyses were conducted using JMP version 13 software (SAS Institute Inc., Cary, NC, USA).

## Results

Table 1 presents the demographic characteristics, namely, gender, age, arch type, tooth site, reasons for extraction, and smoking status, of participants who received dental implants. Overall, 92 dental implants were included in the ARP group and 249 dental implants in the SH group. The percentage of men in the ARP group (*n* = 47; 51.1%) was similar to that in the SH group (*n* = 129; 51.8%). The age at treatment (years, mean ± SD) of the ARP group (53.39 ± 11.97 years) was not statistically significant than that of the SH group (50.94 ± 11.60 years). Neither the frequency of the arch type nor the frequency of the tooth site exhibited any significant intergroup differences. The distributions of tooth extraction caused by periodontitis (*χ*^2^ = 5.25, *p* = 0.02) and smoking (*χ*^2^ = 4.90, *p* = 0.03) exhibited a significant intergroup difference. Table 2 presents the association between a treatment modality and healing patterns of extraction sockets. The treatment of dental implant placement without ancillary bone grafting augmentation procedure was more likely to be used in the ARP group, and bone grafting augmentation procedure via GBR prior to dental implant placement was likely to be used in the SH group. Therefore, a significant association was observed between the treatment modality of bone grafting augmentation procedure via GBR prior to dental implant placement and SH pattern of the extraction site (*χ*^2^ = 15.07, *p* = 0.0005). The effects of healing patterns of the extraction socket on various treatment modalities in clinics as per logistic regression analysis are illustrated in Table 3. Based on the dental implant placement without ancillary bone grafting augmentation procedure treatment modality, the crude risk of bone grafting augmentation procedure via GBR prior to dental implant placement was significantly associated with the SH pattern (OR = 4.65, 95% CI = 2.15–11.61; *p* = 0.0003) than the ARP pattern. After adjustment for gender, age, arch type, tooth site, reasons of extraction, and smoking status using the multivariate logistic regression analysis, the risk of the SH pattern was noted to be still significantly associated with the treatment modality of bone grafting augmentation procedure via GBR prior to dental implant placement (adjusted OR = 5.02, 95% CI = 2.26–12.85; *p* = 0.0002).

**Table 1:** Demographic characteristics of study patients per the healing patterns of alveolar ridge preservation (ARP) and spontaneous healing (SH) of tooth extraction sites

	ARP	SH	*χ*^2^	*p* value^+^
	*n* = 92 (%)	*n* = 249 (%)		
Gender			0.01	0.91
Male	47 (51.1)	129 (51.8)		
Female	45 (48.9)	120 (48.2)		
Age (years, mean ± SD)	53.39 ± 11.97	50.94 ± 11.60		0.09^‡^
Arch type			0.14	0.71
Maxilla	33 (35.9)	84 (33.7)		
Mandible	59 (64.1)	165 (66.3)		
Tooth site			0.04	0.85
Premolar	14 (15.2)	40 (16.1)		
Molar	78 (84.8)	209 (83.9)		
Reasons of extraction			5.25	0.02
Periodontitis	37 (40.2)	68 (27.3)		
Non-periodontitis	55 (59.8)	181 (72.7)		
Smoking status			4.90	0.03
Smoker	6 (6.5)	39 (15.7)		
Non-smoker	86 (93.5)	210 (84.3)		

*p* value were assessed using the chi-square test^+^ and two-sample Student’s *t* test^‡^

**Table 2:** The associations between treatment modality and healing patterns of tooth extraction sockets

	Treatment modality				
	Implant alone *n* = 209 (%)	Simultaneous GBR and implant, *n* = 59 (%)	Staged GBR before implant, *n* = 73 (%)	*χ*^2^	*p* value*
Healing patterns				15.07	0.0005
SH	140 (67.0)	43 (72.9)	66 (90.4)		
ARP	69 (33.0)	16 (27.1)	7 (9.6)		

*p value was analyzed using the chi-square test

*ARP* alveolar ridge preservation, *SH* spontaneous healing

**Table 3:** The effects of healing patterns of extraction sockets on treatment modalities in clinics as per the logistic regression analysis

Healing patterns	Risks of simultaneous GBR and implant				Risks of staged GBR before implant			
	COR (95% CI)	*p* value	AOR^#^ (95% CI)	*p* value	COR (95% CI)	*p* value	AOR^#^ (95% CI)	*p* value
ARP	1.00		1.00		1.00		1.00	
SH	1.32 (0.71–2.58)	0.3911	1.62 (0.78–3.49)	0.2047	4.65 (2.15–11.61)	0.0003	5.02 (2.26–12.85)	0.0002

Reference outcome is implant alone

*SH* spontaneous healing, *ARP* alveolar ridge preservation, *COR* crude odds ratio, *AOR* adjusted odds ratio, *CI* confidence interval

^#^Adjusted by gender, age, arch type, tooth site, reasons for extraction, and smoking status in logistic regression analysis

## Discussion

ARP often maintains adequate bone and soft tissue volume and facilitates simplified treatment during the subsequent dental implant at a prosthetic-driven position. Recently, increasing studies have explored whether additional augmentations are needed at the time of dental implant insertions with the ARP pattern [14, 25, 26]. In a systematic review, Horvath et al. described that only limited evidence supports the clinical benefit of ARP, namely, a reduced need for further augmentation in conjunction with dental implant placement [13]. Weng and Schliephake indicated that the tooth extraction site without ARP had five times increased risk of having alveolar ridge augmentation at dental implant placement than the extraction with ARP [27]. In another meta-analysis, Willenbacher et al. determined that dental implants could be inserted into the determined prosthodontic-driven position without further augmentation in 90.1% of ARP patterns, whereas this was only 79.2% in extraction sockets with the SH pattern [22]. Our study results are in accordance with that of a previous study in that the dental implant placement at the extraction site with SH healing pattern had a 5.02-fold increased risk of having staged GBR compared with the ARP healing pattern. In a systematic review of resorption after the extraction socket, Van der Weijden et al. [6] noted that clinical loss was higher in terms of bone thickness than bone height. The resorption of the alveolar bone causes a narrower and shorter knife-edge ridge [28], often resulting in the tip of the ridge positioning in a more lingual or palatal position relative to the original tooth position [1, 2]. The bone resorption after extraction can lead to a narrower ridge width with inadequate volume for dental implantation. Therefore, without alveolar ridge augmentation to increase the buccal width and coronal height, the dental implant will assume a more lingual or palatal position, resulting in a poor emergence profile or even overhang prosthesis.

Hence, an alveolar ridge augmentation before dental implant treatment is necessary to facilitate a favorable prosthetic position of the dental implant [29, 30]. Notably, the cumulative success rate and survival rate of dental implants in the native bone and regenerative bone were similar [31, 32]. Furthermore, another study revealed that no differences related to marginal bone height and peri-implant soft tissue parameters were observed between dental implants placed with simultaneous bone regeneration and dental implants placed into the native bone [33]. Even though hard tissue augmentation can correct the hard tissue deficiency resulting from extraction [34] around the dental implant and elevate the success rate, survival rate, as well as hard and soft tissue volumes, soft or hard tissue alveolar ridge augmentation procedure entails prolonged treatment duration and higher costs. In addition, to attain tension-free primary closure and avoid early exposure of the membrane and graft materials, flap advancement can reduce the vestibular depth and keratinized mucosa width [35]. Regarding the decrease in keratinized mucosa width, the ARP pattern with non-resorbable membrane [36] or resorbable membrane [26] exhibited better preservation of facial keratinized mucosa width than the SH pattern. Hence, these study results support the fact that the ARP pattern can be a protective factor in reducing the risk of the tooth extraction site having additional alveolar ridge augmentation surgery before dental implant placement.

Notably, anterior teeth dental implants were excluded from this study. A buccal undercut frequently exhibits at anterior teeth. Based on the CBCT examination, Zhang et al. observed that the percentages of buccal undercuts were as follows: central incisor: 41%, lateral incisor: 77%, and canine: 33%, respectively [37]. Moreover, the labial plate in the anterior teeth was thin (0.78–0.97 mm) on CBCT examination [38]. Furthermore, a 20% occurrence rate of fenestration is detected if an anterior dental implant is placed in the cingulum position with the axis after restoration. The depth of the buccal undercut concavity was significantly higher in the fenestration sites (4.79 mm) than in non-fenestration sites (3.40 mm) [39]. Moreover, ARP at the extracted socket provided an ideal outcome. Notably, bone grafting augmentation procedure via GBR prior to dental implant placement or bone grafting augmentation procedure via GBR at the same time of dental implant placement is often needed to increase the apical bone volume at anterior tooth sites. Therefore, to avoid underestimation of the effect of ARP, we evaluated only the effect of ARP at the posterior teeth. Meanwhile, increasing studies found that alveolar ridge preservation at posterior teeth with an intact alveolar socket [40] or non-molar and molar teeth with non-intact alveolar sockets [41] could observe significantly reduced requirement for bone augmentation at implant placement.

This study has some limitations. First, because of data collection from medical records, the time interval from dental extraction to dental implant treatment could not be clearly defined in patients with the SH pattern. In the ARP group, radiographic data and reasons for tooth extraction were clearly labeled in medical records, with the atraumatic extraction and ARP then performed clinically. The time interval from tooth extraction, ARP, pre-implant CBCT evaluation, and dental implant placement was clearly recorded as 4–5 months. However, in the SH group, participants who received dental implant treatment at missing sites were not able to remember the exact date of tooth extraction. Notably, after tooth extraction, the alveolar bone is resorbed most rapidly during the first 6 months, and the subsequent bone resorption continues throughout life at a slower rate [42]. If the extraction occurred 4–5 or more months ago, the alveolar bone resorption is more severe. Participants in the SH group had a prolonged period of missing tooth than did those in the ARP group and hence incurred more bone resorption, as observed on CBCT evaluation. This observation could explain the reason for finding higher frequencies of staged alveolar ridge augmentation before dental implant placement in the SH group with more severe bony destruction than the ARP group. Second, the reasons for tooth extraction in this study were divided into periodontitis and non-periodontitis. Patients with periodontitis probably had a damaged alveolar socket after tooth extraction than non-periodontitis patients. This is another limitation of the study to point out.

Another limitation of this study that was not discussed is the phenotypic characteristics. The extent of bone modeling after extraction depends on the facial bone wall thickness. Whereas thin bone wall phenotypes (< 1 mm) often show a progressive bone resorption and extensive vertical loss of the former socket wall, thick bone wall phenotypes (> 1 mm) show only limited resorption [43]. A facial bone wall thickness of ≤ 1 mm was identified as a critical factor associated with the extent of bone resorption. Thin-wall phenotypes displayed pronounced vertical bone resorption, as compared with thick-wall phenotypes [44]. Despite that other ridge preservation techniques are currently available and their clinical significance has been already demonstrated, only allografts (FDBA) were enrolled in the ARP group. Nevertheless, well-designed, prospective studies can be conducted in the future to compare the effect of ARP patterns by using different types of graft materials, such as autogenous bone, xenografts, and alloplastic grafts.

To the best of our knowledge, this is the first study to investigate the effect of the ARP pattern on treatment modalities of dental implant placement at tooth extraction sites. Nevertheless, further prospective, double-blind, and well-designed research should be conducted with a large sample size involving different types of graft and membrane materials, as well as flap designs of the ARP pattern to ascertain whether additional augmentation procedures can be avoided at dental implant sites, thereby simplifying surgical procedures and reducing the treatment duration.

## Conclusion

The results of this study showed that implant placement without ancillary bone grafting augmentation procedure treatment modality was significantly associated with the ARP pattern at the posterior tooth extraction site. In addition, the dental implant installed at the extraction site with the SH pattern had a 5.02-fold increased risk of having bone grafting augmentation procedure via GBR prior to implant placement compared with the ARP pattern. Hence, additional studies regarding surgical techniques and ARP materials are needed to analyze which treatment is more efficient in preserving the alveolar bone volume at the extraction site and facilitating a clinically efficacious dental implant treatment.
